# Age-related changes of protein SUMOylation balance in the AβPP Tg2576 mouse model of Alzheimer's disease

**DOI:** 10.3389/fphar.2014.00063

**Published:** 2014-04-07

**Authors:** Robert Nisticò, Caterina Ferraina, Veronica Marconi, Fabio Blandini, Lucia Negri, Jan Egebjerg, Marco Feligioni

**Affiliations:** ^1^IRCCS Fondazione Santa LuciaRome, Italy; ^2^Department of Physiology and Pharmacology, Sapienza University of RomeRome, Italy; ^3^Laboratory of Pharmacology of Synaptic Plasticity, EBRI “Rita Levi-Montalcini” FoundationRome, Italy; ^4^Center for Research in Neurodegenerative Diseases, C. Mondino National Neurological InstitutePavia, Italy; ^5^Neuroscience Drug Discovery DKH. Lundbeck A/S, Valby, Denmark

**Keywords:** sumoylation, Tg2576, Alzheimer's disease, SENP1, Ubc9, SUMO-1, SUMO-2/3, neurodegeneration

## Abstract

Alzheimer's disease (AD) is a complex disorder that affects the central nervous system causing a severe neurodegeneration. This pathology affects an increasing number of people worldwide due to the overall aging of the human population. In recent years SUMO protein modification has emerged as a possible cellular mechanism involved in AD. Some of the proteins engaged in the physiopathological process of AD, like BACE1, GSK3-β tau, AβPP, and JNK, are in fact subject to protein SUMO modifications or interactions. Here, we have investigated the SUMO/deSUMOylation balance and SUMO-related proteins during the onset and progression of the pathology in the Tg2576 mouse model of AD. We examined four age-stages (1.5, 3, 6, 17 months old) and observed shows an increase in SUMO-1 protein conjugation at 3 and 6 months in transgenic mice with respect to WT in both cortex and hippocampus. Interestingly this is paralleled by increased expression levels of Ubc9 and SENP1 in both brain regions. At 6 months of age also the SUMO-1 mRNA resulted augmented. SUMO-2-ylation was surprisingly decreased in old transgenic mice and was unaltered in the other time windows. The fact that alterations in SUMO/deSUMOylation equilibrium occur from the early phases of AD suggests that global posttranslational modifications may play an important role in the mechanisms underlying disease pathogenesis, thus providing potential targets for pharmacological interventions.

## Introduction

Alzheimer's disease (AD) is considered one of the most common and debilitating pathologies in the elderly. AD is a slowly progressive neurodegenerative disease that is characterized by impairment of memory and eventually by other symptoms (Heun et al., 2013).

Research indicates that the disease is associated with the production of oligomers of amyloid beta (Aβ) leading to progressive neuritic plaque deposition and hyperphosphorylation of microtubule protein tau with subsequent formation of neurofibrillary tangles (Tiraboschi et al., 2004).

An “oxidative stress hypothesis” for AD has been recently postulated (Markesbery, 1997; Di Domenico et al., 2011; Leitao et al., 2011), albeit it is still unclear whether oxidative stress represents a trigger mechanism to unbalance normal cell functions or might be rather the consequence of pathogenic events. SUMOylation is among the PTMs that has been recently linked to AD (Lee et al., 2013). In fact, SUMOylation induces critical changes on AD-associated proteins like microtubule-associated protein tau (MAPT), amyloid β precursor protein (AβPP) (Georgopoulou et al., 2001; Marcus and Schachter, 2011), c-Jun terminal kinase (JNK) (Feligioni et al., 2011; Sclip et al., 2013) and AMPA receptors (Jaafari et al., 2013), that play an important role in neuronal physiology (Pittaluga et al., 2005, 2006; Holman et al., 2007), therefore providing novel targets for therapeutic intervention. SUMO family includes at least three paralogs (SUMO-1 to -3) ubiquitously expressed in all organism tissues including the brain (Droescher et al., 2013). SUMOs target proteins through non-covalent or covalent interactions affecting their cellular localization, aggregation, metabolism and activity (Steffan et al., 2004; Martin et al., 2007; Feligioni et al., 2011; Krumova et al., 2011; Droescher et al., 2013). Protein SUMOylation has recently been recognized to play a fundamental role in oxidative stress (Bossis and Melchior, 2006; Feligioni et al., 2011; Leitao et al., 2011; Feligioni and Nisticò, 2013), in the regulation of glutamate release (Feligioni et al., 2009) and also in the modification of activity of several intracellular proteins like AβPP and tau (Dorval and Fraser, 2006, 2007; Zhang and Sarge, 2008). As a consequence, SUMO-mediated alterations in specific intracellular signaling pathways could promote AD pathogenesis. Altogether, these observations indicate that SUMOylation could play an important role in the onset of AD, though its precise contribution still remains elusive. Here we performed an age-related analysis on the expression levels of global protein SUMOylation and SUMO-related enzymes in the Tg2576 mouse modeling AD. Importantly, we observe significant differences in SUMO/deSUMOylation balance at an early stage of the pathology.

## Materials and methods

### Ethics statement

All experiments were done in accordance with the European Community Council Directive of 24 November 1986 (86/609/EEC) and approved by the Ethical Committee on animal experiments of EBRI “Rita Levi-Montalcini” Foundation (Rome, Italy).

### Brain tissue dissection

Adult male and female Tg2576 (Brecht et al., 2005) mice were sacrificed by cervical dislocation and immediately after hippocampal and cortical brain regions were dissected on ice. Both Tg2576 and WT mice were processed in parallel and were used for the experiments at the different age stages (1.5, 3, 6, and 17 months). Once removed, brain tissues were immediately placed in a cryopreservation solution {containing 0.32 M sucrose, buffered to pH 7.4 with Tris-(hydroxymethyl)-amino methane [Tris, final concentration (f.c.) 0.01 M]} and stored at −80°C until needed. The cortical and hippocampal tissues of Tg2576 and their wild-type (WT) littermates have been kindly provided by Lundbeck AS (Denmark).

### Preparation of lysate from brain tissue

Around 200 μg of mice tissues were lysed in 300 μl of Lysis Buffer solution (LB) made up of 1% Triton X-100 (Serva, Germany), complete protease inhibitor cocktail solution (Serva, Germany), phosphatase inhibitor cocktail solution (Serva, Germany), 20 mM of NEM (Sigma–Aldrich) and the following components (mM): TRIS acetate, 20; sucrose, 0.27; EDTA, 1; EGTA, 1; Na Orthovanadate, 1; NaF, 50; Na Pyrophosphate, 5; Na β-glycerophosphate, 10; DTT, 1.

Samples were then kept for 30 min on ice to allow protein solubilization. Later a centrifugation step of 10 min at 12000 rpm was applied to the samples and the supernatant was collected and stored at −20°C until needed.

### Western blot

Protein concentrations for each sample were determined by Bradford assay and the samples were directly analyzed by immunoblotting following resuspension in Laemmli buffer.

Equal amount of proteins (~15 μg for each condition) were resolved by 10% SDS-polyacrylamide gels and blotted onto PVDF membrane (Serva, Germany). The proteins blotted on the membrane were then blocked for 1 h at room temperature using Tris-buffered saline-Tween (t-TBS) (M) Tris, 0, 02; NaCl, 0, 15; Tween 20, 0, 1%) containing 5% skimmed milk.

Later the membranes were treated with specific antibodies and the incubation last for 12 h at 4°C with mild agitation. The primary antibody used for western blot analysis are: rabbit amyloid precursor protein (AβPP) 1:2000 (Sigma-Aldrich, USA), rabbit SUMO-1 1:1000 (Cell Signaling, USA), rabbit SUMO-2/3 (18H8) 1:1000 (Cell Signaling, USA), rabbit SENP1 1:500 (Thermo scientific, USA), mouse UBC9 (C12) 1:1000 (Santa Cruz Biotechnology, USA), mouse β-actin 1:30000 (Sigma-Aldrich, Italy).

Tris-buffer saline solution (TBS) with 0, 1% of Tween 20 was used to wash the membranes for 50 min of wash in t-TBS. Then the blots were incubated for 1 h at room temperature with peroxidase-conjugated goat anti-rabbit or anti-mouse IgG secondary antibodies (UCS Diagnostic), as needed.

After 50 min of washes in t-TBS bands immunoreactivity was detected by enhanced chemiluminescence (ECL; WESTAR, Cyanagen, Italy). For all experiments stripping procedure was applied when the control of loading, performed blotting for β-actin, was required (stripping buffer from SignaGen, USA).

For each time point, a western blot has been performed in which proteins from six samples for WT and Tg2576 have been separated and analyzed. Where possible, membranes have been stripped and re-blotted for different antibodies with the purpose of using same loading conditions.

### RNA extraction and real-time PCR

Total RNA was extracted from brain cortex and hippocampus using the Trizol reagent (Invitrogen, Carlsbad, CA) according to the manufacturer's instruction. RNA yield and purity were determined by spectrophotometry absorption at 260 and 280 nm. To obtain cDNA, an equal amount of mRNA (1 μg) underwent to Reverse Transcription (Promega, Madison, WI). The resulting cDNA was stored at −20°C until used for the further analysis. Messenger RNA expression was quantitatively measured with quantitative (q) real time PCR using iCycler Bio-Rad. The reaction was performed in a 25 μl volume using SensiMix SYBR Green and Fluorescein kit (Bioline, London, UK). All the measures were performed in triplicate. The reaction conditions were as follows: 95°C for 10 min (Polymerase activation), followed by 40 cycles at 95°C for 15, 55–50°C (Temp. depends on the Tm of primers) for 15 s and 72°C for 15 s. The reaction mixture without the cDNA was used as control.

The primer sequences used in this study were as following for SUMO-1: forward 5'-GCCTGGGACATGGGTTT-3' and reverse 5'-TTAATGAAGCTGGTACAGACGATG-3'; SUMO-2: forward 5'-GGCAGGGTTTGTCAATGAGGC-3' and reverse 5'-CTGGAGTAAAGTA GTAGCAGGCTC-3'; SUMO-3: forward 5'-GAGGCAGGGCTTGTCAATGAG-3' and reverse 5'-GGTCAGGACAACGGTTGGGTG-3'; SENP1: forward 5'-AATGGCTGATGATGATGTG-3' and reverse 5'-TTGGACAAGGATTAGACTGAAT-3'; UBC9: forward 5'-CATCCAGCCTTCGT AAACC-3' and reverse 5'-GCTAACAGGCAGGGAGAT-3'; glyceralde-hydes-3-phosphate dehydrogenase (GAPDH): forward 5'-GCCAAGGCTGTGGGCAAGGT-3' and reverse 5'-TCTCCAGGCGGCACGTCAGA-3'.

The *Ct* values of the specific gene of interest were normalized to the *Ct* value of the endogenous control, GAPDH, and the comparative *Ct* method (2^−ΔΔ*Ct*^) was then applied using WT mice group as calibrator.

### Statistical analysis

In the western blots and real time PCR experiments, *T*-test analysis was performed and *p* < 0.05 was considered statistically significant. Statistical analysis for biochemical experiments was performed using GraphPad PRISM 5. A number (as indicated in figure legends) of animal tissues has been used at the same time point for the experiments. Values shown represent the mean ± s.e.m. Final western blots have been run using representative sampling

## Results

### AβPP increases in Tg2576 mice

The Tg2576 transgenic mouse carries a transgene coding for the 695-amino acid isoform of human AβPP derived from a large Swedish family with early-onset AD (Hsiao et al., 1996). This mouse model of AD expresses high concentrations of mutant Aβ, develops a significant number of amyloid plaques, and displays functional deficits like decreased dendritic spine density, impaired long-term potentiation (LTP), and behavioral deficits (Jacobsen et al., 2006; Balducci et al., 2011).

**Figure 1:**
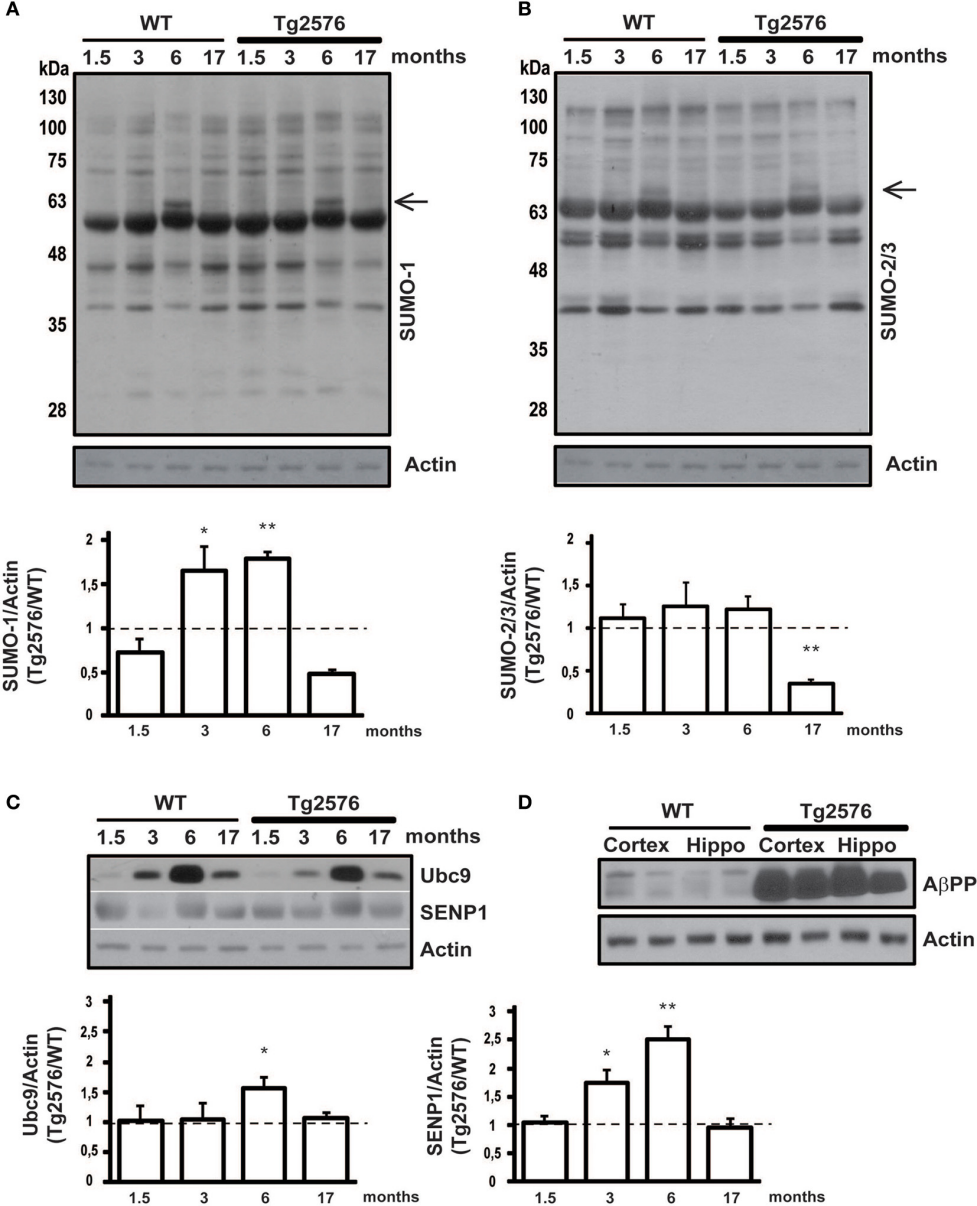
**Protein SUMOylation and SUMO-related proteins expression level in cortical tissue.** Representative western blots of WT and Tg2576 mice cortical tissue lysate. Cortex of WT and Tg2576 animals was analyzed at four time points (1.5, 3, 6, and 17 months of age). Anti-SUMO-1 **(A)**, anti-SUMO-2/3 **(B)**, anti-Ubc9, anti-SENP1 **(C)**, and anti-AβPP **(D)** have been used for the protein detections. Results are expressed as ratio between Tg2576 and WT. Data represent means ± s.e.m. of 6 animals for each age. (*t*-test) ^**^p < 0.01 or ^*^p < 0.05 Tg2576 vs. WT. β-Actin was used as loading control.

In order to validate the mice model used in this work we measured AβPP immunoreactivity in the cortical and hippocampal tissues from the brains of Tg2576 and WT mice. Tg2576 are expected to have an augmented expression of AβPP because, beside the murine endogenous AβPP, they genetically over-express human mutated AβPP. Indeed, both cortex and hippocampus prepared from Tg2576 mice showed a more intense immunoreactivity of the AβPP band corresponding to 95–100 kDa compared to WT (Figure 1D).

### Protein SUMOylation changes in the cortex of Tg2576 mice during ontogenesis

The cerebral cortex is among the most vulnerable brain regions affected by the disease showing progressive plaque burden and tissue atrophy (Tosun et al., 2011). Although in a less severe manner, some biochemical hallmarks have also been reported in different AD mice models (Sclip et al., 2011; Izco et al., 2014).

In order to evaluate protein SUMOylation changes during disease progression, we dissected and lysated cortical tissues from 1.5, 3, 6, 17 months old Tg2576 and their age-matched WT littermates. Protein SUMOylation has been revealed by western blotting using an anti-SUMO-1 and an anti-SUMO-2/3 antibody. SUMO-1 and SUMO2/3 for each time point has been normalized against the actin. Interestingly, the ratio in protein SUMO-1-ylation between Tg2576 and WT mice is increased at early stages, and it later decreases following a bell-shaped curve. In fact, a peak of SUMO-1-ylation is observed at 3 and 6 months old animals (Figure 1A, 3 months: 1.65 ± 0.16 *p* < 0.05; 6 months: 1.78 ± 0.08 p < 0.01).

Noteworthy a protein SUMOylated band, absent in the other ages analyzed, appears at 6 months of age. The immunoreactivity of this band results more intense in the Tg2576 mice compared to controls (Figure 1A, black arrow). Further studies are required to identify which protein becomes target of SUMO-1 at this stage, and whether this protein modification has a role in AD pathology.

SUMO-2/3-ylation did not show any differences in the ratio between Tg2576 and WT mice at different ages except for 17 months old mice, where it resulted drastically decreased in transgenic mice (Figure 1B, 17 months: 0.35 ± 0.05 p < 0.01). Further experiments are required to understand what implication this reduction may have. As for the SUMO-1 western blot, also for SUMO-2/3 a more intense band is detectable (Figure 1B, black arrow).

### Protein SUMOylation changes in the hippocampus of Tg2576 mice during ontogenesis

The hippocampus is the part of the brain that is involved in memory formation, being one of the first regions to suffer damage in AD. Since Tg2576 show cognitive impairment caused by hippocampal dysfunction even before the onset of the pathology (D'Amelio et al., 2011), we analyzed protein SUMOylation changes between Tg2576 and WT mice also in this brain area.

**Figure 2:**
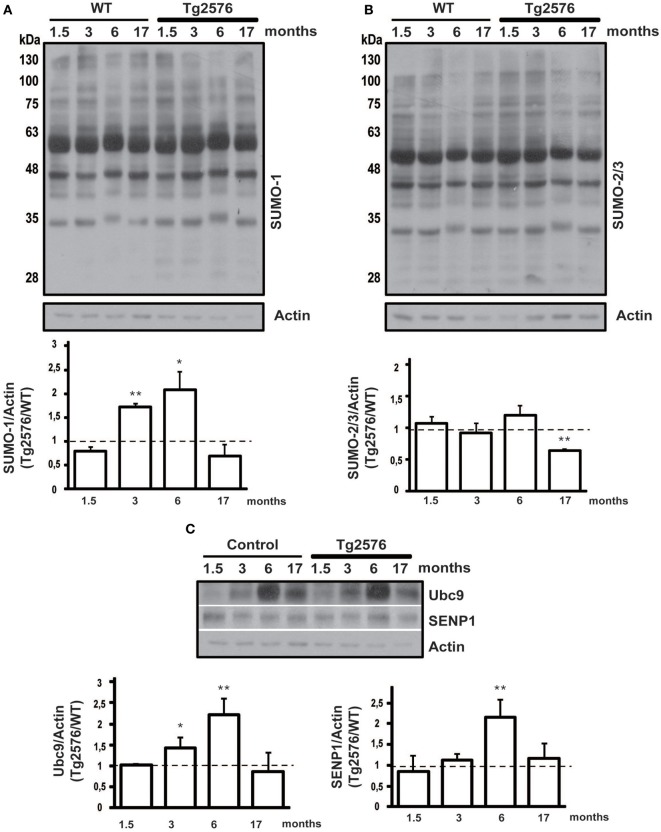
**Protein SUMOylation and SUMO-related proteins expression level in hippocampal tissue.** Representative western blots of WT and Tg2576 mice hippocampal tissue lysate. Hippocampus of WT at four time points (1.5, 3, 6, and 17 months of age) was compared with their age-matched Tg2576 mice. Anti-SUMO-1 **(A)**, anti-SUMO-2/3 **(B)**, anti-Ubc9 and anti-SENP1 **(C)** have been used for protein detections. Results are expressed as ratio between Tg2576 and WT. Data represent means ± s.e.m. of 6 animals for each age. (*t*-test) ^**^p < 0.01 or ^*^p < 0.05 Tg2576 vs. WT. β-Actin was used as loading control.

Similarly to the cortex, SUMO-1-ylation is increased in the hippocampus of 3 and 6 months old Tg2576 compared to WT (Figure 2A, 3 months: 1.71 ± 0.07 p < 0.01; 6 months: 2.07 ± 0.39 *p* < 0.01).

SUMO-2/3-ylation did not show any differences in the ratio between Tg2576 and WT mice at different ages except for 17 months old, where surprisingly it resulted decreased in transgenic mice (Figure 2B, 17 months: 0.65 ± 0.01 p < 0.01). This result needs more investigation in order to understand its potential implication in the pathophysiology of AD.

### SUMO pathway related proteins in Tg2576 transgenic mice ontogenesis

The protein conjugation by SUMO occurs through the activity of four key enzymes which form the SUMOylation pathway. Among them Ubc9 and SENP1 are the most important since they are dedicated exclusively to protein SUMOylation. On the one hand Ubc9 binds and transports SUMO to the target protein, on the other SENP1 is the isopeptidase that regulates the deSUMOylation (Droescher et al., 2013; Feligioni and Nisticò, 2013). Ubc9 and SENP1 are critical for the maintenance of SUMO/deSUMOylation balance, therefore their expression levels have been assessed by western blotting in cortical and hippocampal tissue from Tg2576 and WT mice. The expression level of the two proteins has been normalized on actin and the ratio between Tg2576 and WT has been analyzed.

Interestingly, we found that the expression level of Ubc9 is modulated both in cortical and hippocampal tissue. In fact, the western blots show for both WT and Tg2576 an increase of Ubc9 from 3 to 17 months with a peak at 6 months of age. Moreover Ubc9 seems to be highly expressed in Tg2576 compared to WT at different ages (Figure 1C, 6 months: 1.57 ± 0.16 *p* < 0.05) (Figure 2C, 3 months: 1.42 ± 0.24 p < 0.05; 6 months: 2.22 ± 0.37 *p* < 0.01). Conversely, immunoreactivity of SENP1 seems to be augmented in the cortex of transgenic mice at 3 and 6 months and only at 6 months in hippocampus of Tg2576 compared to age-matched WT animals (Figure 1C, 3 months: 1.75 ± 0.21 *p* < 0.05; 6 months: 2.51 ± 0.21 p < 0.01) (Figure 2C, 6 months: 2.15 ± 0.42 p < 0.01).

### SUMO-1-, SUMO-2-, SUMO-3-, SENP1- and Ubc9-mRNA in brain cortex and hippocampus

**Figure 3:**
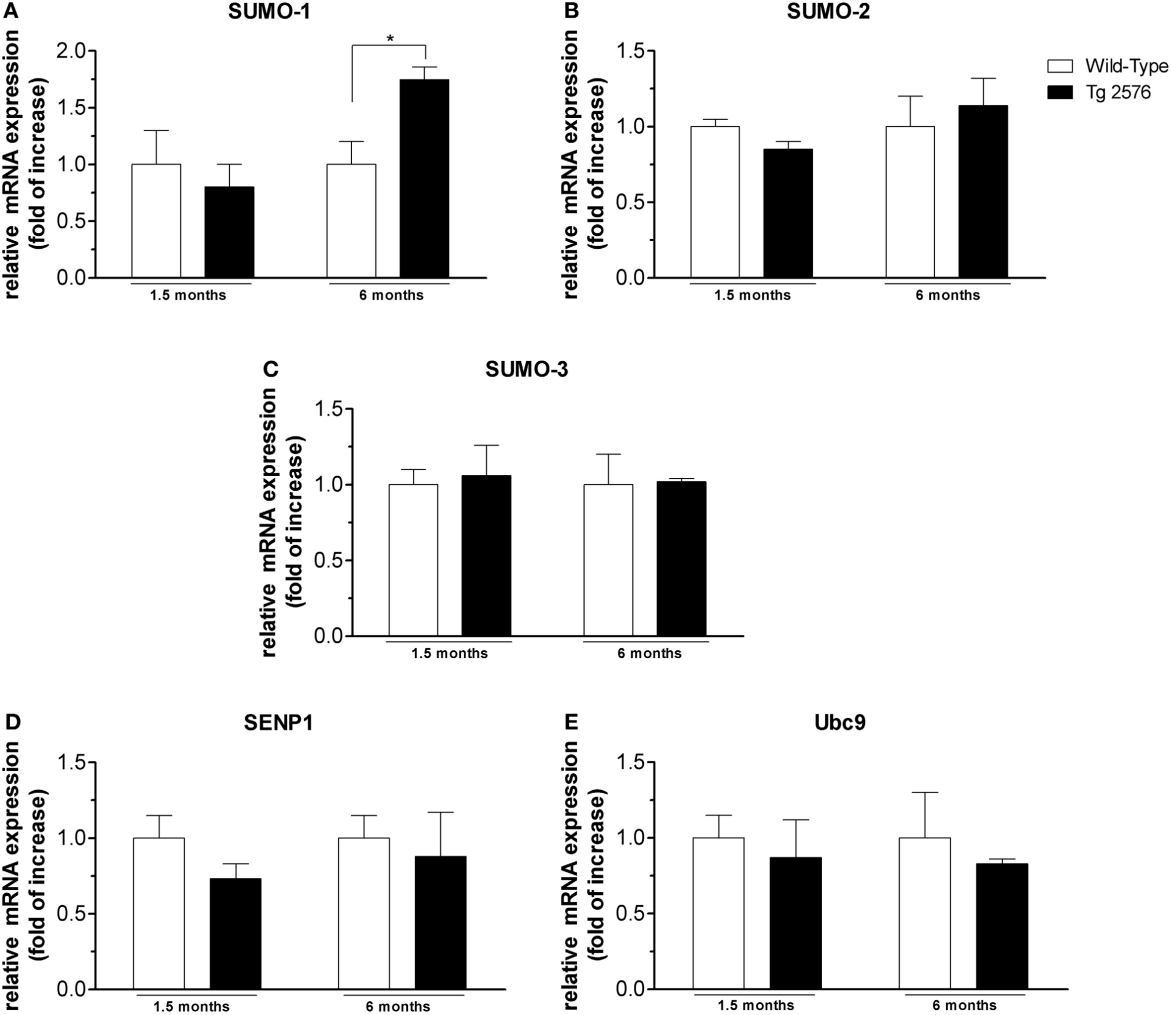
**mRNA expression level of SUMO and SUMO-related proteins in cortical tissues.** Analyses of SUMO-1 **(A)**, SUMO-2 **(B)**, SUMO-3 **(C)**, SENP1 **(D)**, Ubc9 **(E)** mRNA expression in brain cortex of Tg2576 and WT mice at the age of 1.5 and 6 months. The mRNA expression levels, determined by real-time polymerase chain reaction (RT-PCR), were expressed in relation to glyceraldehyde 3-phosphate dehydrogenase (GAPDH) and presented as fold increase relative to WT animals. Data represent mean ± s.e.m. of 4 animals. (*t*-test) ^*^p < 0.05 Tg2576 vs. WT.

**Figure 4:**
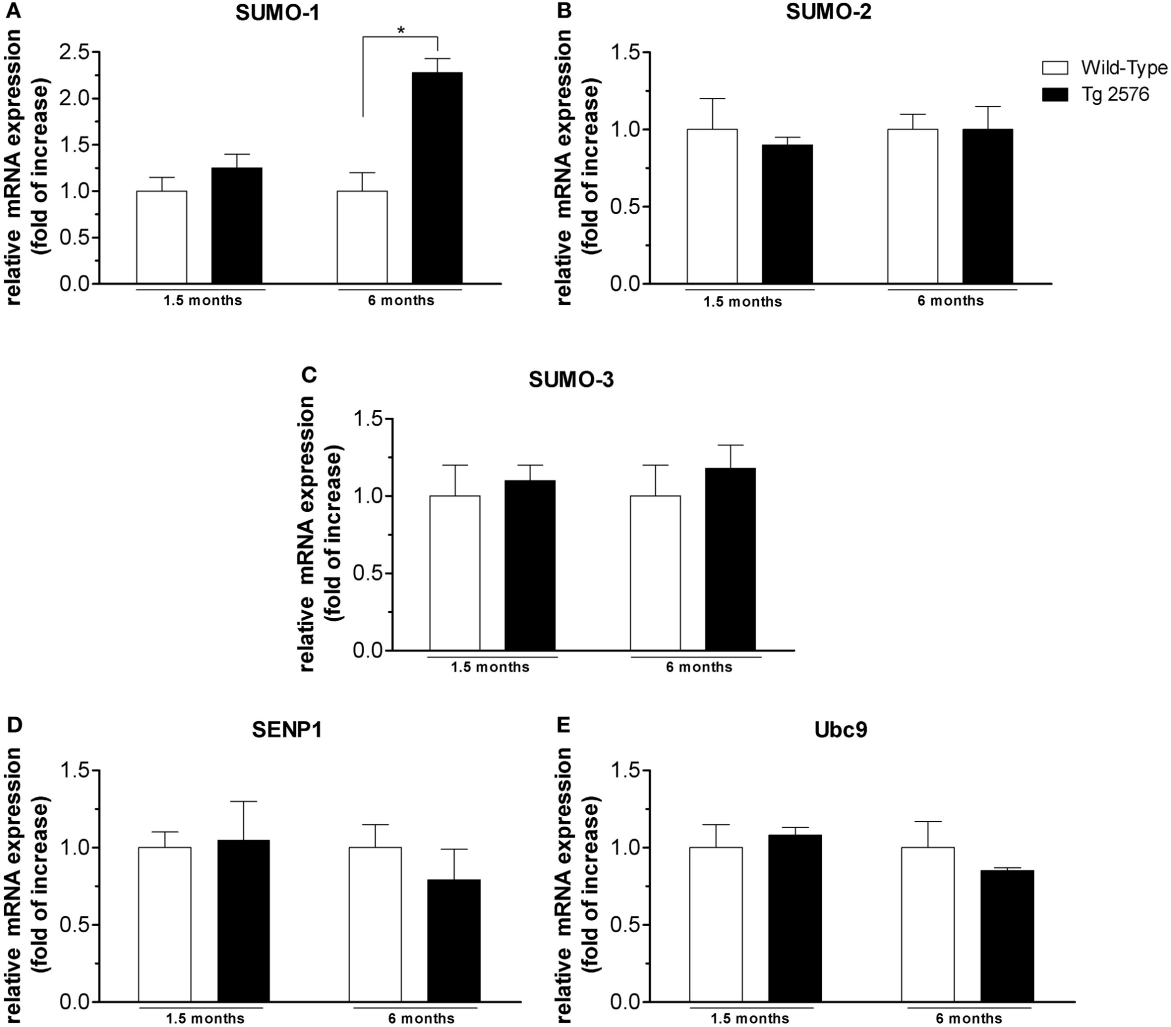
**mRNA expression level of SUMO and SUMO-related proteins in hippocampal tissues.** Analyses of SUMO-1 **(A)**, SUMO-2 **(B)**, SUMO-3 **(C)**, SENP1 **(D)**, Ubc9 **(E)** mRNA expression in brain hippocampus of Tg2576 and WT mice at the age of 1.5 and 6 months. The mRNA expression levels, determined by real-time polymerase chain reaction (RT-PCR), were expressed in relation to glyceraldehyde 3-phosphate dehydrogenase (GAPDH) and presented as fold increase relative to WT animals. Data represent mean ± s.e.m. of 4 animals. (*t*-test) ^*^p < 0.05 Tg2576 vs. WT.

SUMO-1 mRNA levels in the cortex (Figure 3A) and hippocampus (Figure 4A) of Tg2576 mice at 1.5 months of age were similar to the levels measured in WT mice, whereas at 6 months of age their levels were significantly higher than that of WT mice. On the contrary, we did not find any significant changes in SUMO-2, SUMO-3, SENP1, and Ubc9 mRNA levels in the cortex (Figures 3B–E) and hippocampus (Figures 4B–E) of Tg2576 and WT mice at the age of 1.5 and 6 months. The SUMO-1 mRNA increase is in accordance with the elevated level of SUMO-1-ylation that has been found in cortex and hippocampus at 6 months of age. It seems that SUMO-1 synthesis is augmented in order to be disposable for conjugation, whereas the Ubc9 and SENP1 protein levels are sufficient in both tissues to maintain SUMOylation pathway functional. The SUMO-2 and -3 mRNA level is in line with protein SUMO-2/3-ylation expression that resulted unchanged up to 6 months of age.

## Discussion

Tg2576 mice model were developed to test the amyloid hypothesis of AD. These mice model answered partially the question whether AD pathology comes from the accumulation of Aβ species. Aβ species, although poorly detectable, can be found in the soluble form (monomers, dimers, trimmers or oligomers) already at a young age (<5 months old) (Kawarabayashi et al., 2001; Klingner et al., 2003; Lesné et al., 2006), but they reach a highly detectable level around 6–8 months (Hsiao et al., 1996). Insoluble amyloid plaques start to form at around 9–10 months in hippocampus and cortex (Hsiao et al., 1996). This model also shows the typical AD cognitive impairment with a mild onset at 3 months, which is exacerbated starting from 6 months of age. However, Tg2576 do not resemble all the features of AD since they do not display any formation of neurofibrillary tangles, marked neuronal loss or gross brain atrophy, (Irizarry et al., 1997), but are anyway a very useful model to study AD amyloid-related pathology (Deacon et al., 2008).

Several AD-associated proteins undergo SUMO protein modification, leading to hypothesize that SUMO plays a fundamental role in AD pathogenesis. Despite that, we still lack information about how SUMO/deSUMOylation process is altered during AD onset and development. Therefore a better understanding of SUMO changes during AD pathogenesis could help to disclose the role of protein modification in the onset of AD. Notably, McMillan and co-authors (McMillan et al., 2011) reported that no clear differences in global protein SUMOylation can be measured in adult Tg2576 (9 months old) vs. their respective WT.

In this manuscript, we decided to investigate SUMOylation changes during mice lifespan analyzing four time points. To our knowledge this is the first ontogenetic study of SUMO/deSUMOylation profile in an AD model.

Yun and co-authors have shown in *in-vitro* experiments that SUMO-1 can be considered a modulator of the synthesis of Aβ oligomers (Yun et al., 2013). In fact SUMO-1, but not SUMO-2 or -3, when overexpressed in neuronal or cell culture, increases BACE1 level which mediates the amiloydogenic cleavage of AβPP. Moreover, as previously reported in 18 months old AβPP transgenic mice (Yun et al., 2013) SUMO-1 free protein is increased in their cortical tissue. So, it has been postulated that the increase of Aβ accumulation together with an accrual of cell oxidative stress can act synergistically to enhance protein SUMO-1-ylation. By conjugating BACE1, SUMO-1 intensifies Aβ oligomers production (Yun et al., 2013). Our data show that already at the age of 3 months, protein SUMO-1-ylation is augmented both in cortical and hippocampal tissue of Tg2576 model compared to their respective WT.

Therefore it can be hypothesized that at early stages (3 months) the “cell stress” might contribute more to the increase in SUMO-1-ylation compared to Aβ oligomers which are present at low levels at this age. As a result, SUMO-1-ylation activates multiple signaling pathways, like BACE1, GSK3β, JNK activation, that can contribute to boost the production of Aβ oligomers. The feedback loop established between SUMO-1, Aβ oligomers and cell stress cooperate to the development of AD neuropathology. Interestingly, SUMO-2-ylation does not change between Tg2576 and WT except for 17 months old transgenic mice where it is drastically diminished both in cortex and hippocampus. This aspect also deserves a deeper investigation since very little is known about SUMO-2/3 contribution to AD.

Ubc9 and SENP1 are critical enzymes for protein SUMOylation (Droescher et al., 2013; Feligioni and Nisticò, 2013). An increase of SUMOylation is associated with the onset of several pathologies including cancer. For example, Ubc9 expression, the E2 enzyme which facilitates SUMOylation, has been reported increased in primary colon and prostate cancer compared with normal tissue (Moschos et al., 2010). We here demonstrate that the increase of SUMOylation in Tg2576 brain tissues corresponds to a high expression level of Ubc9 at the same time points. Therefore Ubc9 plays a fundamental role for SUMOylation event also in AD. On the other hand, the over-functionality of SUMOylation in Tg2576 is also supported by an elevated presence of the SUMO protease SENP1. Our results are in line with previous data where SENP-1 was found increased during oxygen-glucose deprivation (OGD) experiments, suggesting that the neuronal response could involve a complex interplay between SUMOylation and deSUMOylation (Cimarosti et al., 2012).

The analysis of mRNA expression has shown an increase of SUMO-1 RNA only at the age of 6 months both in cortex and hippocampus. This is in line with our western blot data where the major increase of SUMOylation was exactly found at 6 months of age in Tg2576 mice, where probably there is a need for more protein synthesis. In contrast, although we reported an increase in protein expression level of both Ubc9 and SENP1, the mRNA expression of both proteins was unchanged. We can therefore speculate that the degradation system of Ubc9 and SENP1 could be decreased during lifespan of mice. Further studies should be carried out in order to better clarify this aspect. In line with a previous work (McMillan et al., 2011), our experiments did not show significant changes in protein SUMO-2/3-ylation in AD mice up to 6 months of age. SUMO-2/3 role in AD remains still elusive. However, in *in-vitro* experiments it has been shown that SUMO-2 increases N-terminal ADAM-cleaved AβPP fragment (α-NTF) while a mutant form of SUMO-2 lacking the SUMOylation activity secreted significantly more N-terminal BACE1-cleaved AβPP fragment (β-NTF) and Aβ (Li et al., 2003). In line with this report, we can speculate that the reduction in protein SUMO-2/3-ylation that was observed in 17 months old Tg2576 mice is concomitant with an elevated amyloid deposition which occurs at this stage in AD mice model (Jacobsen et al., 2006). In conclusion, here we report for the first time that protein SUMO/deSUMOylation equilibrium is unbalanced in a mouse model of AD at a very early stage of the pathology. We believe that this event can contribute to the onset of AD pathogenesis. Future investigation on the target(s) of protein SUMOylation at an early stage could possibly lead to the identification of novel pharmacological targets.

### Conflict of interest statement

The authors declare that the research was conducted in the absence of any commercial or financial relationships that could be construed as a potential conflict of interest.
